# Dr. William C. Fritz

**Published:** 1903-05

**Authors:** 


### OBITUARY.

Dr. William C. Fritz, of Buffalo, U. of B., '92, died suddenly March 31, 1903, aged 36 years. He had been ill for about three months with rheumatism and his heart became involved. He was thought to be convalescing and called upon Dr. Eugene A. Smith, who was ill, on the morning of the day of his death. While in conversation with Dr. Smith, Dr. Fritz, sank in his chair, gasped for breath and in an instant more was dead. Dr.

Fritz was regarded as one of the rising younger physicians of this city, and was instructor in clinical surgery at the University of Buffalo. He is survived by a widow and three children.

